# Supplementary material for: Non-diffusive slow heat dissipation induces high local temperature in living cells
Source: Nat Commun. 2026 May 28;17:4215. doi: 10.1038/s41467-026-71878-y (PMC13219429; doi:10.1038/s41467-026-71878-y)
Supplement: Supplementary file 2 — Description of Additional Supplementary Files [file 41467_2026_71878_MOESM2_ESM.pdf]

Supplementary Movie 1

Movie of Rhodamine B fluorescence inside single liposome upon heating with IR laser.

Supplementary Movie 2

Movie of fluorescence lifetime of FPT inside single COS7 cell upon heating with IR laser.

Supplementary Movie 3

Movie of fluorescence lifetime of FPT inside single COS7 cell upon heating for 500 ms with IR laser.

Supplementary Movie 4

Movie of fluorescence lifetime of FPT inside single COS7 cell upon heating for 1 s with IR laser.

Supplementary Movie 5

Movie of fluorescence lifetime of FPT inside single COS7 cell upon heating for 5 s with IR laser.

Supplementary Movie 6

Movie of fluorescence lifetime of FPT in the cytoplasmic region of COS7 cells upon heating with IR laser.

Supplementary Movie 7

Movie of fluorescence lifetime of FPT in the nuclear region of COS7 cells upon heating with IR laser.
